# Evaluation of the antiplasmodial properties of selected plants in southern Ethiopia

**DOI:** 10.1186/s12906-015-0976-x

**Published:** 2015-12-23

**Authors:** Solomon Asnake, Tilahun Teklehaymanot, Ariaya Hymete, Berhanu Erko, Mirutse Giday

**Affiliations:** Medicine and Health Science College, Hawassa University, P.O. Box 1560, Hawassa, Ethiopia; Aklilu Lemma Institute of Pathobiology, Addis Ababa University, P.O. Box 1176, Addis Ababa, Ethiopia; School of Pharmacy, Addis Ababa University, P.O. Box 1176, Addis Ababa, Ethiopia

**Keywords:** Antimalarial plants, *Plasmodium berghei*, Sidama people, Ethiopia

## Abstract

**Background:**

The majority of the Ethiopian population is at risk of malaria largely caused by *Plasmodium falciparum.* The resistance of the parasite to existing drugs is the main challenge in the control of the disease and thus new therapeutic drugs are required. In Ethiopia, people use different plant species to treat malaria. However, very few of them have so far been evaluated for their safety level and antimalarial activity. Thus, the aim of this study was to evaluate the safety and antimalarial activity of extracts of *Ajuga integrifolia*, *Clerodendrum myricoides*, *Melia azedarach*, *Peponium vogelii* and *Premna schimperi*, locally used by the Sidama people of Ethiopia to treat malaria.

**Methods:**

The safety level of 80 % methanol extracts of the plants were evaluated using standard acute toxicity test procedure. The antiplasmodial activity of 80 % methanol extracts of the plants were assessed in vivo using Swiss albino mice against chloroquine sensitive rodent malaria parasite, *Plasmodium berghei*, using the standard 4-day suppressive test procedure at doses of 200,400 and 800 mg/kg/day. The 80 % methanol extract of *Ajuga integrifolia* that exhibited better antimalarial activity was fractionated using different solvents and screened for its phytochemical constituents and evaluated in vivo for its antimalarial activity at doses of 100, 200 and 400 mg/kg/day.

**Results:**

All extracts given at the three different doses caused no lethal effect on mice in 24 h and within 10 days of observation. All extracts and fractions exhibited antimalarial activity in a dose dependant manner. The highest inhibition was exhibited by the crude extracts of *A. integrifolia* (35.17 %) at 800 mg/kg/day (*P* < 0.05). Among fractions of *A. integrifolia*, n-butanol fraction demonstrated the highest inhibition (29.80 %) at 400 mg/kg/day (*P* < 0.05). The extracts and fractions prolonged the survival time and prevented weight loss of the mice, but did not prevent PCV reduction. Phytochemical test on *Ajuga integrifolia* indicated the presence of alkaloids, flavonoids, saponins, terpenoids, anthraquinone, steroids, tannins, phenols and fatty acids.

**Conclusions:**

Findings show that the plants are non-toxic and demonstrate antimalarial activity in a dose dependant manner suporting claims of their traditional therapeutic value for malaria treatment. However, further in-depth investigation is required to assess the potential of the plants towards the development of new antimalarial agent.

## Background

Malaria continues to be a leading cause of morbidity and mortality in sub-Saharan African countries, especially among children under the age of five and pregnant women [1]. Besides its major public health problem, the disease also has negative impact on socioeconomic development. Malaria mostly affects people in productive age groups and causes substantial economic loss because of the compromised capacity and efficiency of the labour force [2].

Malaria is a major public health problem in Ethiopia; more than 60 % of the Ethiopian population is at risk of malaria and approximately 62 % of malaria cases are due to *P. falciparum* [3]. Malaria was the leading cause of morbidity and mortality in the years 2002, 2003 and 2004 [4]. However, since 2005 the incidence of malaria and death in the country has declined due to scale up of intervention strategies together with the administration of artemisin combination therapy (ACT) [5]. However, the parasite is less sensitive to antimalarial drugs such as chloroquine, amodiaquine and sulphadoxine pyrimethamine [6]. There are also reports on parasite resistance to currently existing first line drug regimen ACT in parts of Cambodia and Thailand [7] as well as reduced sensitivity in parts of Africa [8]. Such reports necessitate a search for new and effective antimalarial drug.

Traditional medicinal remedies are viable treatment alternatives for communities that lack access to available drugs and they provide an opportunity to introduce novel antimalarials too. Medicinal plants served as source of two major antimalarial drugs, quinine from Peruvian medicinal plant *Cinchona succirubra* tree bark and artemisinin from Chinese medicinal plant *Artemisia annua* [9].

In Ethiopia, there is a widespread use of traditional medicine among urban and rural population, which might be related to accessibility, cultural acceptability and economic affordability of the system as compared to modern medicine [10]. Investigations carried out in the country indicated that traditional healers and indigenous people in different parts of the country utilize different species of medicinal plants [11–15] for treatment of malaria. There are still more other medicinal plants that are claimed to be effective by local communities in Ethiopia but not documented and evaluated for their safety level and antimalarial activity. Thus, the purpose of this study was to evaluate the antiplasmodial properties of *Ajuga integrifolia*, *Clerodendrum myricoides*, *Melia azedarach*, *Peponium vogelii* and *Premna schimperi* used by the Sidama people, southern Ethiopia and fraction of *Ajuga integrifolia*. The five plants were the ones that had the highest relative frequency of citation (RFC) as revealed from an ethnobotanical study conducted in Boricha District, Sidama Zone of the South Region of Ethiopia by the same investigators involved in the current study. According to Trotter and Logan [16], plants that are used in some repetitive fashion are more likely to be biologically active. Previous studies conducted on the plants indicated their antiplasmodial activity [17–19]. An in vivo study conducted on water extract of *Ajuga remota*, a close relative of *A. integrifolia* treated mice showed 90.4 % parasitaemia suppression at a dose of 30 μg/mL [17]. Aqueous, methanol and dichloromethane extracts of the root bark of *C. myricoides* exhibited in vitro antimalarial activity with IC_50_ values of 64 μg/mL, 48.2 μg/mL, 15. μg/mL, respectively [18]. A study conducted on bark extract of *M. azedarach* revealed an in vitro antimalarial activity with IC_50_ value of 66.2 μg/mL [19]. Study carried out on crude methanol extract and fractions of petroleum ether, dichloromethane and ethyl acetate of the bark of *Premna angolensis*, relative species of *P. schimperi*, indicated antimalarial activity with IC_50_ values of 180, 250, 250 and 250 μg/mL, respectively [20]. Phytochemical screening conducted on *C. myricoides* showed the presence of secondary metabolites such as saponins, phytosteroides, polyphenols, flavonoides, tannins and alkaloids [21].

## Methods

### Plant collection

For the in vivo test, samples of *Ajuga integrifolia*, *Clerodendrum myricoides*, *Melia azedarach*, *Peponium vogelii* and *Premna schimperi* were collected from Boricha District, southern Ethiopia, during one of the field trips to the area. Specimen of each plant was dried, identified at Aklilu Lemma Institute of Pathobiology and National Herbarium of Addis Ababa University and voucher deposited at the National Herbarium.

### Preparation of crude extracts

Parts of the plant samples were shade dried, ground using electric grinding mill (Laboratory mill, Arthuur A. Thomas company Philadelphia, USA), sieved by using sieve number 85, weighed using electronic balance and placed in plastic bags until extraction. One hundred gram of each powdered medicinal plant part was extracted with 500 mL of 80 % methanol using a soxhlet extractor [22]. Then, methanol was removed using rotary evaporator at a temperature of about 40–45 °C and the water part was removed using freeze dryer (lyophilizer). The dried extract of each plant part was collected in labeled airtight small bottles and kept in deep freezer until used.

### Fractionation of crude extracts

*Ajuga integrifolia*, a plant with better antimalarial effect, was further fractionated using solvent fractionation technique [23]. Sequential solvent partitioning of the 80 % methanol crude extract was conducted to get different solvent fractions. Twenty grams of the extract was suspended in 350 mL of distilled water in a separatory funnel. The aqueous portion was partitioned three to four times with 100 mL of chloroform to obtain chloroform fraction. Then, aqueous residue was further fractionated three to four times with 100 mL of n-butanol to obtain n-butanol fraction. Finally, the aqueous solution was collected as the third fraction. The chloroform and n-butanol fractions were concentrated in a rotary evaporator. The aqueous fraction was frozen in refrigerator overnight and then, dried using a lyophilizer. The fractions obtained were put in airtight bottles and stored in a refrigerator at 4 °C until used.

### Phytochemical screening

Phytochemical screening to test for the presence of secondary metabolites (alkaloids, terpenoids, flavonoids, tannins, saponins, steroids, anthraquinon and phenols) and proteins, carbohydrates, and fats and oils in the crude extract of *A. integrifolia* was carried out using standard procedures [24].

### In vivo acute toxicity test

Acute toxicity test of the extracts was conducted in Swiss albino male mice of six to eight weeks of age and weighing 25–30 g. The test was performed by randomly dividing 30 mice into six groups of five mice per cage. The mice were fasted over night and groups 1 to 5 received 2000 mg/kg of the extract, whereas control (group 6) mice received the vehicle, distilled water [25]. Then, the mice were observed for any gross behavioral change and death one hour after the treatment, intermittently for four hours, and thereafter over a period of 24 h. The mice were further observed for 10 days.

### In vivo antimalarial test

The test was carried out based on the four-day suppressive test described by Peters et al. [26]. Male Swiss albino mice weighing 25–30 g were put randomly into test and control groups, each group containing five mice and were supplied with adequate amount of mouse cubes and clean drinking water. The plasmodium strain used for the test was *P. berghei*, a chloroquine sensitive rodent malaria parasite, obtained from the Drug Research Department of the Ethiopian Public Health Institute. The parasitized erythrocytes for each test were collected from an infected donor mouse with rising parasitaemia of 20–30 %. The mice were sacrificed by head blow, and blood was collected in a Petri dish with an anticoagulant (0.5 % trisodium citrate) by severing the jugular vein.

The blood was then diluted with physiological saline (0.9 %) in proportion of 1:4. Each mouse was then inoculated with 0.2 mL of blood containing about 10^7^*P. berghei* infected erythrocytes on day 0 through intra peritoneal route. After three hours of parasite inoculation, three test groups of mice were administered with 200, 400 and 800 mg/kg of the crude extracts. Similarly, in fraction treated mice, three test groups of infected mice were treated with 100, 200 and 400 mg/kg of the fractions. The negative control group mice were treated with 0.2 mL of the vehicle (distilled water) and the positive control groups were treated with 10 mg/kg of chloroquine.

### Determination of parasitaemia

On the fifth day, a drop of blood was taken from tail snip of each mouse on frosted slide and smears were prepared, fixed with methanol and stained with 10 % Giemsa solution at pH 7.2 for 15 min. Then, five fields were randomly selected on each stained slide and examined under microscope with an oil immersion objective (×100 magnification power). The parasitaemia level was determined by counting the number of parasitized erythrocytes on randomly selected fields of the slide. Percentage of parasitaemia and suppression was calculated using the formulas [27] given below.

$$ \begin{array}{l}\%\ \mathrm{Parasitaemia}=\frac{\mathrm{Number}\ \mathrm{of}\ \mathrm{parasitized}\ \mathrm{RB}\mathrm{C}\mathrm{x}100}{\mathrm{Total}\ \mathrm{number}\ \mathrm{of}\ \mathrm{R}\mathrm{B}\mathrm{C}\ \mathrm{counted}}\hfill \\ {}\%\ \mathrm{of}\ \mathrm{suppression}=\frac{\left(\mathrm{Parasitaemia}\ \mathrm{in}\ \mathrm{negative}\ \mathrm{control}\hbox{--} \mathrm{Parasitaemia}\ \mathrm{in}\ \mathrm{treated}\ \mathrm{group}\right)\mathrm{x}100}{\mathrm{Parasitaemia}\ \mathrm{in}\ \mathrm{negative}\ \mathrm{control}}\hfill \end{array} $$

### Determination of mean body weight and survival time

The body weight was determined by taking average weight of mice in each test group and comparing it with that of infected negative controls. Mortality was monitored daily and the number of days from the time of inoculation of the parasite up to death was recorded for each mouse in the treatment and control groups throughout the follow up period. The mean survival time (MST) for each group was calculated as given below.

$$ \mathrm{M}\mathrm{S}\mathrm{T}=\frac{\mathrm{Sum}\ \mathrm{of}\ \mathrm{survival}\ \mathrm{time}\ \left(\mathrm{days}\right)\ \mathrm{of}\ \mathrm{mice}\ \mathrm{in}\ \mathrm{group}}{\mathrm{Total}\ \mathrm{numbers}\ \mathrm{of}\ \mathrm{mice}\ \mathrm{in}\ \mathrm{that}\ \mathrm{group}} $$

### Determination of the packed cell volume (PCV)

Blood was collected from sniped tail of each mouse in heparinized microhaematocrit capillary tubes. Each capillary tube was sealed by crystal seal and placed in a micro-hematocrit centrifuge (Hettich haematokrit, Germany) with the sealed ends out wards. Blood that was filled to three fourth of the capillary tubes was centrifuged at 12,000 rpm for 5 min. The volume of the total blood and the volume of erythrocytes were measured and PCV was calculated using the formula:

$$ \mathrm{P}\mathrm{C}\mathrm{V}=\frac{\mathrm{Volume}\ \mathrm{of}\ \mathrm{R}\mathrm{B}\mathrm{C}\ \mathrm{in}\ \mathrm{a}\ \mathrm{givenblood}\ \mathrm{volume}\mathrm{x}\ 100}{\mathrm{Total}\ \mathrm{blood}\ \mathrm{volume}} $$

### Data analysis

The collected data were organized, entered into Microsoft Office Excel 2007 and exported to Windows SPSS 20.1. Frequencies and percentages were calculated from the data using the Excel 2007. Data on parasitaemia, survival day, body weight and packed cell volume were analyzed using Windows SPSS version 20.1. One way ANOVA and paired sample *t*-test were used to compare results within groups for difference between initial (before) and final (after) treatments. Results obtained from the study were presented as mean plus or minus standard error of the mean (M ± SEM). All data were analyzed at 95 % confidence interval (α =0.05).

## Results

### Percentage yield of extracts

The yield of the extracts obtained from the five antimalarial plants was in the range of 10–50 g. The highest percentage of yield was obtained from *A. integrifolia* (30.0 %) and the lowest from *P. schimperi* (18.0 %) (Table 1). The physical nature of the extracts showed that crude extracts of leaf and areal parts were semi solid (gumy), while extracts from bark were solid.

**Table 1:** Yield of 80 % methanol extracts of medicinal plant

Scientific name	Sidama name	Habit	Part used	% yield
*Ajuga integrifolia*	Anamuro	Herb	Aerial	30.0
*Clerodendrum myricoides*	Madisisa	Herb	Leaf	20.0
*Melia azedarach*	Mime	Tree	Twig	24.7
*Peponium vogelii*	Surupa	Climber	Leaf	27.3
*Premna schimperi*	Udo	Shrub	Leaf	18.0

### Acute toxicity test of crude extracts

Acute toxicity test conducted to determine the safety level of crude extracts of the five plants showed that the lethal dose of all extracts was above 2000 mg/kg. All extracts that were administered orally in a single dose of 2000 mg/kg to the laboratory bred Swiss albino mice caused no lethal effect within 24 h of the observation period. The gross behavioral and physical observation of the experimental mice also revealed that the extracts created no visible signs of acute toxicity such as lacrimation, hair erection and reduction in their motor and feeding activities. The mice were physically active and fed and drunk as that of the control groups administered with the vehicle (distilled water) within the observation period of 10 days.

### In vivo antimalarial test of crude extracts

Result of the four-day suppressive test of the crude extracts of the five antimalarial plants at different dose levels on mice infected with *P. berghei* expressed as percent of reduction of parasitaemia in reference to the negative control mice is summarized in Table 2. The result indicated that all the crude extracts of the five antimalarial plants exhibited antiplasmodial activity. The extracts reduced parasitaemia to different levels in dose dependant manner. The highest inhibition was exhibited by crude extract of *A. integrifolia* (35.17 ± 1.95) at 800 mg/kg/day and the lowest by *Premna schimperi* (2.27 ± 0.07) at 200 mg/kg/day.

**Table 2:** Effect of 80 % methanol crude extract of medicinal plants on parasitaemia level of *P. berghei* infected Swiss albino mice

Medicinal plant	Dose mg/kg/day	D4 post-infection		*P* value
		% parasitaemia (M ± SEM)D4	% suppression (M ± SEM)	
*Ajuga integrifolia*	200	42.56 ± 1.80^*a^	21.06 ± 1.27	0.000
	400	40.49 ± 1.40^*a^	24.87 ± 1.54	0.000
	800	34.92 ± 1.20^*a^	35.17 ± 1.95	0.000
*Clerodendrum myricoides*	200	52.19 ± 1.13	3.88 ± 1.36	0.851
	400	49.95 ± 1.60	7.18 ± 1.94	0.166
	800	45.78 ± 1.49^*a^	14.99 ± 2.02	0.001
*Melia azedarach*	200	50.96 ± 1.50	5.43 ± 1.50	0.462
	400	42.95 ± 1.60^*a^	21.75 ± 1.31	0.000
	800	36.84 ± 1.22^*a^	31.54 ± 2.18	0.000
*Peponium vogelii*	200	54.48 ± 1.06	2.62 ± 0.89	0.789
	400	53.23 ± 0.89	5.54 ± 0.60	0.337
	800	51.99 ± 0.85	7.20 ± 0.77	0.074
*Premna schimperi*	200	56.70 ± 0.83	2.27 ± 0.70	0.933
	400	55.90 ± 0.64	2.63 ± 0.31	0.486
	800	54.78 ± 0.65	4.56 ± 0.58	0.066
NC	0	53.84 ± 0.83	0.00 ± 0.00	
PC	10	0.00 ± 0.0	100 ± 0.00	

Values are expressed as M ± SEM; *n* = 5

*NC* negative control (0.2 mL of dH_2_O); *PC* positive control (chloroquine), *D4* day four

^*^ *P* < 0.05

^a^compared to negative control

Comparison of each test group to their respective negative control groups at different dose level indicated that the crude extracts of *A. integrifolia* reduced parasitaemia to significant level on day 4 (*p* < 0.05) at doses of 200, 400 and 800 mg/kg. The crude extract of *M. azedarach* also reduced parasitaemia to significant level on day 4 (*p* < 0.05) at doses of 400 and 800 mg/kg. In addition, extract of *C. myricoides* reduced parasitaemia significantly on day 4 only at dose of 800 mg/kg (*p* < 0.05). Parasite reduction induced by extracts of *P. schimperi* and *P. vogelii* was not significant (*P* > 0.05) at all the three doses.

### Determination of mean survival time of extract treated mice

Result of the survival time of mice treated with different doses of the 80 % methanol extract (Table 3) indicated that treated mice survived for more days than the respective negative control groups. Moreover, the highest survival time was recorded in mice that received the highest dose of extracts. Among extract-treated mice, those treated with 800 mg/kg/day of crude extract of *A. integrifolia* survived for longer time with mean survival time of 12.00 ± 0.44. Mice that were treated with crude extract of *P. schimperi* survived for shorter time with mean survival days (7.4 ± 0.24) at oral dose of 200 mg/kg/day.

**Table 3:** Effect of 80 % methanol crude extracts of medicinal plants on mean survival time of *P. berghei* infected Swiss albino mice

Medicinal plant	Dose mg.kg/day	Mean survival time (M ± SEM)	*P* value
*A. integrifolia*	200	9.40 ± 0.24^*a^	0.000
	400	9.80 ± 0.38^*a^	0.000
	800	12.00 ± 0.44^*a^	0.000
*C. myricoides*	200	8.20 ± 0.20	0.141
	400	9.00 ± 0.31^*a^	0.015
	800	10.00 ± 0.39^*a^	0.002
*M. azedarach*	200	8.60 ± 0.24^*a^	0.032
	400	9.30 ± 0.37^*a^	0.011
	800	10.90 ± 0.58^*a^	0.001
*P. vogelii*	200	7.80 ± 0.20	0.454
	400	8.20 ± 0.24	0.227
	800	9.20 ± 0.37^*a^	0.017
*P. schimperi*	200	7.40 ± 0.24	0.307
	400	8.00 ± 0.31	0.084
	800	9.40 ± 0.50^*a^	0.030
NC	0	6.40 ± 0.24	
PC	10	30.00 ± 0.00	

Values are expressed as M ± SEM; *n* = 5

*NC* negative control (0.2 mL of dH_2_O), *PC* positive control (Chloroquine)

* = *P* < 0.05

a = compared to negative control

Comparison of the mean survival time of each test group with the respective negative controls at different dose level indicated that the crude extracts of *A. integrifolia* and *M. azedarach* prolonged the mean survival time of mice significantly (*P* < 0.05) at doses of 200, 400 and 800 mg/kg/day. Extract of *C. myricoides* prolonged the survival time of mice significantly (*P* < 0.05) at doses of 400 and 800 mg/kg/day. Extracts of *P. schimperi* and *P. vogelii* prolonged the survival time of mice significantly (*P* < 0.05) only at the highest dose of 800 mg/kg.

### Determination of mean weight of extract treated mice

In general, the mean weight of mice that received 80 % methanol extracts (Table 4) increased on day 4 post infection as compared to that of mice on day 0. The weight of mice increased dose dependently. Mice that were given the highest dose showed more weight increment than mice that received the lowest dose. The highest percentage of weight change value was recorded for mice treated with 800 mg/kg/day of *A. integrifolia* extract (10.58 ± 0.68). While the least was recorded for mice that received 200 mg/kg/day of crude extracts of *P. schimperi* (2.10 ± 0.24).

**Table 4:** Effect of 80 % methanol crude extracts of medicinal plants on body weight of *P. berghei* infected Swiss albino mice

Medicinal plant	Dose mg/kg/day	Body weight (M ± SEM)		% of change (M ± SEM)	*P* value
		Pre (D0)	Post (D4)		
*A. integrifolia*	200	29.52 ± 0.29	31.30 ± 0.44	6.01 ± 0.51^*a^	0.000
	400	27.60 ± 0.14	29.08 ± 0.32	8.56 ± 0.83^*a^	0.000
	800	26.86 ± 0.28	29.76 ± 0.51	10.58 ± 0.68^*a^	0.000
*C. myricoides*	200	26.06 ± 0.21	27.34 ± 0.12	4.18 ± 0.60^*a^	0.006
	400	28.82 ± 0.28	30.20 ± 0.14	5.84 ± 0.75^*a^	0.000
	800	25.94 ± 0.15	27.98 ± 0.23	7.55 ± 0.67^*a^	0.000
*M. azedarach*	200	25.56 ± 0.11	26.70 ± 0.09	4.49 ± 0.38^*a^	0.000
	400	26.78 ± 0.18	28.52 ± 0.30	6.56 ± 0.63^*a^	0.000
	800	28.98 ± 0.26	31.32 ± 0.59	8.78 ± 0.89^*a^	0.000
*P. vogelii*	200	26.64 ± 0.18	27.52 ± 0.23	2.29 ± 0.39	0.062
	400	28.60 ± 0.31	29.88 ± 0.30	4.48 ± 0.32^*a^	0.037
	800	28.18 ± 0.31	30.02 ± 0.54	6.52 ± 0.95^*a^	0.008
*P. schimperi*	200	26.70 ± 0.41	27.46 ± 0.48	2.1 ± 0.24	0.065
	400	29.24 ± 0.25	30.14 ± 0.15	4.0 ± 0.31^*a^	0.031
	800	29.38 ± 0.28	31.10 ± 0.33	5.4 ± 0.50^*a^	0.042
NC	0	25.56 ± 0.24	24.10 ± 0.36	−5.56 ± 0.72	
PC	10	26.52 ± 0.50	29.56 ± 0.49	10.73 ± 0.50	

Values are expressed as M ± SEM; *n* = 5

*NC* negative control (0.2 mL of dH_2_O), *PC* positive control (Chloroquine), *D4* day four

*P < 0.05

^a^compared to negative control

Moreover, the comparison of mean weight of extract treated mice to respective controls at each dose level indicated that extracts of *A. integrifolia, C. myricoides* and *M. azedarach* prevented weight loss significantly (*P* < 0.05) at dose of 200, 400 and 800 mg/kg. Wheareas, the weight loss prevention of mice treated with extracts of *P. schimperi* and *P. vogelii* was not significant (*P* > 0.05) at 200 mg/kg but significant (*P* < 0.05) at 400 and 800 mg/kg as compared to the respective negative control of mice treated with distilled water.

A paired sample *t*-test comparison between day 0 (pre treatment) and day 4 (post treatment) indicated that weight increment of mice treated with extracts of *A. integrifolia* and *M. azedarach* was statistically significant (*P* < 0.05) at doses of 200, 400 and 800 mg/kg/day on day 4. In mice treated with extract of *C. myricoides*, the body weight increment of mice was significant (*P* < 0.05) at doses of 400 and 800 mg/kg. While in mice treated with extracts of *P schimperi* and *P. vogelii*, the weight increased significantly (*P* < 0.05) only at a dose of 800 mg/kg/day.

### Determination of mean packed cell volume of extract treated mice

**Fig. 1:**
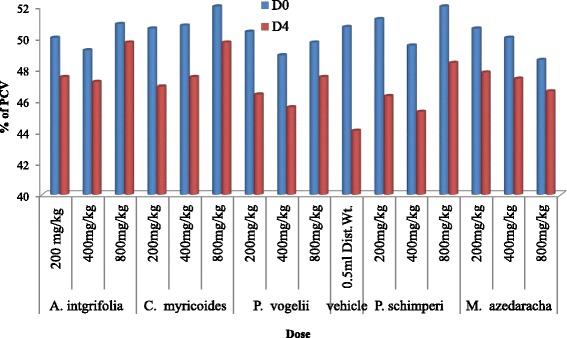
Effect of 80 % methanol crude extracts of medicinal plants on PCV of *P. berghei* infected Swiss albino mice. Keys: Vehicle (NC) = negative control; D0 = day 0; D4 = day 4; PCV = Packed Cell Volume; (n = 5)

The mean value of the packed cell volume (PCV) showed reduction in mice treated with extract (Fig. 1) and in those that were treated with the vehicle on D4 (post treatment) as compared to D0 (pre treatment). However, result of comparison between D0 and D4 of extract treated mice with the respective negative control groups showed no appreciable reduction.

The comparison of PCV change of extracts treated mice at different dose levels with the respective negative controls treated with the vehicle indicated that the PCV value of mice treated with crude extracts of *A. integrifolia* and *M. azedarach* deviated significantly from the negative controls (*P* < 0.05) at doses of 200, 400 and 800 mg/kg/day. The deviation of PCV value of mice treated with extract of *C. myricoides* was significant (*P* < 0.05) at doses of 400 and 800 mg/kg/day. Whereas, in the case of mice treated with extracts of *P. schimperi* and *P. vogelii,* the deviation was significant (*P* < 0.05) only at the highest dose (800 mg/kg). The paired sample *t*-test result between D0 and D4 of mice treated with extracts at doses of 200, 400 and 800 mg/kg/day indicated significant decrease (*P* < 0.05) of PCV value on D4.

### Percentage yield of fractions of *Ajuga integrifolia*

The highest percentage of yield was obtained from n-butanol fraction (35.0 %) of the aerial part of *A. integrifolia*, followed by that of water (24.7 %) and chloroform (17.0 %). There was also variation in the physical appearance of the fractions. The water fraction was sticky and solid while that of n-butanol and chloroform fractions were jelly.

### Phytochemical tests on extract of *Ajuga integrifolia*

Result of phytochemical screening tests on the extract of *A. integrifolia* revealed the presence or absence of main secondary metabolites and other phytochemicals based on presence or absence of expected colour changes (Table 5). The methanol extract of *A. integrifolia* contained alkaloids, terpenoids, flavonoids, steroids, saponins, tannins, anthraquinone, phenols and fats and oils.

**Table 5:** Phytochemical constituents of 80 % methanol extract of *A. integrifolia*

Test	Reagents	Test result
Alkaloid	Wagner’s reagent	++
Terpenoids	Chloroform, acetic anhydride, concentrated sulphuric acid	++
Flavonoids	Magnesium ribbon, concentrated hydrochloric acid	++
Steroids	Chloroform, concentrated H2SO4	+
Tannin	10 % ferric chloride (FeCl_3_)	+
Saponins	distilled water	++
Anthraquinone	KOH	+
Phenol	ferric chloride	+
Proteins	Millions reagent	−
Carbohydrate	Fehling solution, HCl, NaOH	−
Fats and oils	Filter paper	+

+++ = very strong positive, ++ = strong positive, + = fair positive, ^*_*^ = absent

### In vivo antimalarial tests of fractions of *Ajuga integrifolia*

Result of the four-day suppressive test of the three fractions at different dose levels on parasitaemia level of mice infected with *P. berghei* is summarized in Table 6. The result is expressed as the percent of reduction of parasitaemia in reference to the negative control mice treated with the vehicle. As the result indicated, the three fractions exhibited antiplasmodial activity in vivo against *P. berghei*. The fractions reduced parasitaemia to different levels in dose-dependant manner. The highest inhibition of parasitaemia was exhibited by n-butanol fraction (29.80 ± 0.66) at 400 mg/kg/day and the lowest by chloroform fraction (3.68 ± 0.83) at 100 mg/kg/day. The comparison of the test groups to their respective negative controls indicated that the n-butanol fraction reduced parasitaemia to significant level on day 4 (*p* < 0.05) at the doses of 100, 200 and 400 mg/kg/day. Water fraction reduced parasitaemia to significant level (*p* < 0.05) at doses of 200 and 400 mg/kg/day. Parasite reduction of chloroform fractions was not significant (*p* > 0.05) at all the three doses.

**Table 6:** Antimalarial activity of solvent fractions of *A. integrifolia* on parasitaemia level of *P. berghei* infected Swiss albino mice

Fraction type	Dose mg/kg/day	D4 post infection		*P* value
		% of Parasitaemia (M ± SEM)	% of inhibition (M ± SEM)	
Water	100	43.20 ± 1.65	9.72 ± 1.27	0.091
	200	42.40 ± 1.50^*a^	11.29 ± 1.01	0.035
	400	36.60 ± 1.21^*a^	23.44 ± 1.10	0.001
n-Butanol	100	42.20 ± 1.06^*a^	11.80 ± 2.09	0.026
	200	39.80 ± 1.28^*a^	17.16 ± 0.94	0.014
	400	35.80 ± 1.28^*a^	29.80 ± 0.66	0.000
Chloroform	100	47.00 ± 1.41	3.68 ± 0.83	0.752
	200	46.20 ± 1.49	5.78 ± 11.69	0.494
	400	47.80 ± 1.39	9.80 ± 3.41	0.157
NC	0	49.20 ± 1.50	0.00 ± 0.00	
PC	10	0.00 ± 0.00	100.00 ± 0.00	

Values are expressed as M ± SEM; *n* = 5

*NC* negative control (0.2 mL of dH2O), *PC* positive control (Chloroquine), *D4* day four

*P < 0.05

^a^compared to negative control

Although the extracts and fractions reduced parasite load in treated group of mice, none of the crude extracts or fractions completely cleared the parasite. In the positive control group of mice treated with standard antimalarial drug chloroquine phosphate, at daily dose of 10 mg/kg body weight, the parasite was totally cleared on day four-post infection.

### Determination of mean survival time of fraction treated mice

**Fig. 2:**
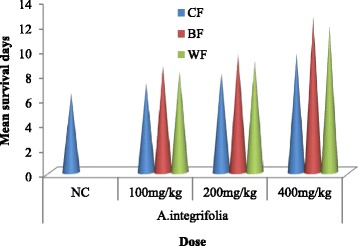
Effect of butanol, water and chloroform fractions of *A. integrifolia* on mean survival time of *P. berghei* infected Swiss albino mice. (*n* = 5). Keys: BF = butanol fraction; WF = water fraction; CF = chloroform fraction; NC = negative control

Result of the mean survival time of mice treated with different doses of the fractions of *A. integrifolia* (Fig. 2) indicated that treated mice survived for more days as compared with those in the negative control group with the highest survival time recorded for mice that received the highest dose of n-butanol fraction (400 mg/kg/day). Comparison of the mean survival time of fraction treated mice to the negative controls at different dose levels showed that the mean survival time of mice that were treated with n-butanol fraction was prolonged significantly (*P* < 0.05) at doses of 100, 200 and 400 mg/kg/day. Water fraction prolonged the survival time of mice significantly (*P* < 0.05) at doses of 200 and 400 mg/kg/day. While, chloroform fraction prolonged the mean survival time of mice significantly (*P* < 0.05) at oral dose of 400 mg/kg/day.

### Determination of mean weight of fraction treated mice

In general, the mean weight of mice that received fractions of *A. intefrifolia* (Table 7) increased on day 4 post infection as compared to that of day 0. The weight of mice increased dose dependently. Mice that were given the highest dose showed more increment than mice that received the lowest dose. Among treated groups, the highest weight change (9.40 ± 0.58) was recorded for mice treated with 400 mg/kg/day of n-butanol fraction and the least (2.26 ± 0.32) for mice that received 100 mg/kg of chloroform fraction. Moreover, the comparison of mean weight of fraction treated mice to respective controls at each dose level indicated that n-butanol and water fractions prevented weight loss of mice significantly at 100, 200 and 400 mg/kg/day doses (*P* < 0.05). Whereas, chloroform fractions prevented weight loss significantly only at 400 mg/kg/day (*P* < 0.05) as compared to the respective negative control mice. A paired sample *t*-test comparison between D0 and D4 indicated significant (*P* < 0.05) weight increment of mice treated with n-butanol and water fractions at dose of 100, 200 and 400 mg/kg/day.

**Table 7:** Effect of water, butanol and chloroform fractions on body weight of *P. berghei* infected Swiss albino mice

Fraction type	Dose mg/kg/day	Body weight (M ± SEM)		% of change (M ± SEM)	*P* value
		D0	D4		
Water	100	26.20 ± 0.44	27.06 ± 0.46	3.28 ± 0.24^*a^	0.027
	200	28.14 ± 0.39	29.82 ± 0.48	6.10 ± 0.30^*a^	0.018
	400	28.80 ± 0.17	31.28 ± 0.13	9.03 ± 0.32^*a^	0.000
n-Butanol	100	27.64 ± 0.09	28.80 ± 0.20	3.19 ± 0.51^*a^	0.033
	200	27.96 ± 0.06	29.38 ± 0.10	6.23 ± 0.48^*a^	0.026
	400	28.52 ± 0. 13	30.40 ± 0.21	6.59 ± 0.58^*a^	0.000
Chloroform	100	26.34 ± 0.25	26.94 ± 0.31	2.26 ± 0.32	0.080
	200	27.12 ± 0.31	28.04 ± 0.39	3.67 ± 0.46	0.072
	400	29.08 ± 0.27	30.58 ± 0.4	5.14 ± 0 .42^*a^	0.031
NC	0	26.80 ± 0.33	25.86 ± 0.27	−2.41 ± 0. .92	
PC	10	27.38 ± 0.51	28.94 ± 0.62	5.00.95 ± 0.71	

Values are expressed as M ± SEM; *n* = 5

*NC* negative control (0.2 mL of dH_2_O), *PC* positive control (Chloroquine)

*P < 0.05

^a^compared to negative control

### Determination of mean packed cell volume of fraction treated mice

**Fig. 3:**
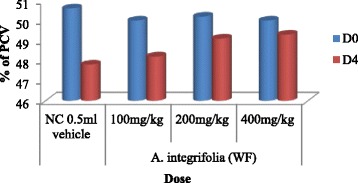
Effect of water fraction of *A. integrifolia* on PCV of *P. berghei* infected Swiss albino mice. Keys: WF = water fraction; NC = negative control; D0 = Day 0; D4 = Day 4; PCV = Packed Cell Volume; (*n* = 5)

**Fig. 4:**
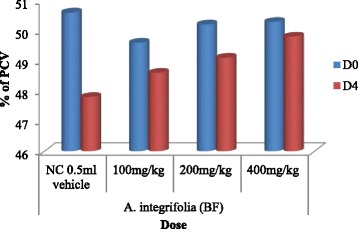
Effect of butanol fraction of *A. integrifolia* on PCV of *P. berghei* infected Swiss albino mice. Keys: BF = butanol fraction; NC = negative control; D0 = Day 0; D4 = Day 4; PCV = Packed Cell Volume; (*n* = 5)

**Fig. 5:**
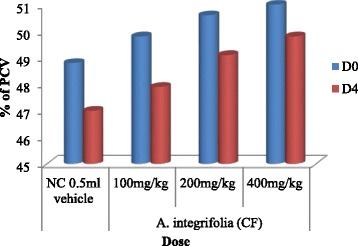
Effect of chloroform fraction of *A. integrifolia* on PCV of *P. berghei* infected Swiss albino mice. Keys: CF = chloroform fraction; NC = negative control; D0 = Day 0; D4 = Day 4; PCV = Packed Cell Volume; (*n* = 5)

The mean value of the packed cell volume (PCV) showed reduction in mice treated with fractions of *A. integrifolia* and those in the negative control group on D4 as compared to D0 (Fig. 3, Fig. 4 and Fig. 5). However, result of comparison of PCV of D0 and D4 fraction treated mice with that of the respective PCV of mice in the negative control group showed no appreciable reduction. The comparative test indicated that PCV values of mice that were treated with n-butanol and water fractions deviated significantly (*P* < 0.05) at doses of 100, 200 and 400 mg/kg/day from that of the mice in the negative control group. The deviation of the PCV value of mice treated with chloroform fraction from that of mice in the negative control group was significant (*P* < 0.05) only at dose of 400 mg/kg/day. The paired sample *t*-test result between D0 and D4 of mice treated with water, n-butanol and chloroform fractions at doses of 100, 200 and 400 mg/kg/day indicated that on day 4, the PCV value showed significant reduction in all test group mice(*P* < 0.05).

## Discussion

As findings of the acute toxicity test indicate, no gross behavioral changes such as impaired movement, reduced motor activity and mortality were manifested in mice that were treated with single dose of 2000 mg/kg. The methanol extract treated mice were in the same condition as that of the control group mice that were treated with distilled water over the observation time of ten days. Thus, the extracts of the tested medicinal plants parts could be considered safe at the dose levels used in this the experiment. The result, therefore, justify the use of such plant parts by the Sidama people for the treatment of malaria. Toxicity tests carried out elsewhere on extracts of the plants *Melia azedarach* [28], *C. myricoides* [29] and *Ajuga* spp. [30] also showed their safety on laboratory animals at doses of 2000 mg/kg and above.

The methanol extracts of the five medicinal plants reduced the malaria parasite load in mice during the four-day suppressive test suggesting their suppressive effect on the blood stage of the parasite. Among the extracts, parasite load was decreased significantly in mice treated with extract of *A. integrifolia* at all the three doses (200, 400 and 800 mg/kg/day). The highest suppression was recorded for *A. integrifolia* at the highest test dose of 800 mg/kg/day. An investigation carried out elsewhere also indicated high level (90.4 %) of suppression of parasitaemia in mice treated with water extract of *Ajuga remota*, a close relative of *A. integrifolia,* at a dose of 30 mg/kg [17]. A study by Irungu et al. [18] revealed that aqueous, methanol and dichloromethane extracts of the root bark extracts of C*. myricoides* exhibited antimalarial activities in vitro with IC_50_ values of 64 μg/mL, 48.2 μg/mL, 15.8 μg/mL, respectively. Bark extract of *Melia azedarach* was reported to show in vitro antiplasmodial activity with IC50 value of 66.2 μg/mL [19].

However, the extracts did not eradicate the parasite completely unlike the standard chloroquine drug. As day-to-day observation during post treatment period indicated, clinical symptoms of the infection were seen in the treated mice due to gradual increment of the parasitaemia. The subsequent development of the parasitaemia in all the extracts treated mice might suggest that the actions of the extracts were short-lived due to their rapid metabolism or elimination [31]. The limited effect also might be related to partial loss of active ingredients due to its insufficient uptake to physiologically active level [32].

Although the mechanism of action of the extracts was not explained, the reduction of parasite load in the treated mice might be due to the presence phytochemical constituents such as alkaloids, flavonoids, and terpenoids. Previous studies have indicated the potential of alkaloids, terpenoids, flavonoids, coumarins, phenolics, polyacetylenes, xanthones, quinones, steroids and lignans for antimalarial drug development [33, 34]. *Ajuga remota*, a related species of *A. integrifolia*, was reported to have shown antimalarial activity due to the presence of terpenoids [33]. Another report also indicated the antimalarial activity of the compounds triterpenoids and limonoids [35].

In vivo evaluation of the n-butanol, water and chloroform fractions of *A. integrifolia* indicated that the n-butanol and water fractions of the plants suppressed parasitaemia to a significant level at all the three doses (100, 200 and 400 mg/kg/day). The relatively high antimalarial action of the n-butanol fraction could be the result of a single or synergistic effect of the secondary metabolites such as alkaloids, saponins, flavonoids, tannins and phenols found in the fraction [36]. The result of the present study is in agreement with findings documented by Yared et al. [37] and Mengiste et al. [38], where n-butanol fraction of *A. africanus* and *D. angustifolia* significantly suppressed parasitaemia in treated mice. The water fraction also reduced parasitaemia dose dependently but the level of activity was less as compared to that of n-butanol, indicating the difference in the type and concentration of the different secondary metabolites in the fractions [39]. The insignificant parasitaemia reduction by chloroform fraction may be due to less concentration of secondary metabolites available in the extract.

Comparison of the activity of the crude extract of *A. integrifolia* with that of its fractions showed that the fractions (n-butanol, water and chloroform) demonstrated less antiplasmodial activity. The relatively higher potency of the crude extract may be attributed to the presence of various phytochemical constituents that work singly or synergistically, but might be reduced or lost during fractionation. Some secondary metabolites protect other metabolites (as antioxidants) and breaking of this association could accelerate degradation and consequently reduction of the suppressive effect of the ingredients [40]. Similar finding was reported indicating greater chemossuppression induced by water extracts of *Ageratum conyzoides* as compared to that of methanol, water and chloroform [41]. Eighty percent methanol leaf extract of *Otostegia integrifolia* also showed to have high antimalarial activity than its respective ethanol, water and chloroform fractions [42].

Treatment with the plants crude extracts and fraction extended survival time of mice in the treatment groups as compared with the mice in non-treated control groups. The survival time of the extract and fraction treated mice for more days in the presence of the parasite might be due to the phytochemical ingredients, which have antioxidant property that reduce the overall pathologic effect of the parasite and prolong the survival time of mice as compared to those in the negative control group. Phytochemical constituents such as flavonoids and tannins have been suggested to act as primary antioxidant or free radical scavengers that aid antioxidant defense system and reduce oxidative stress that is induced by the malaria parasite [43].

Body weight loss is one of the features observed in rodents infected with malaria parasite due to poor appetite that develops because of the intensity of the infection [44]. During the period of 4-day suppressive test, the extracts and fractions protected mice from losing weight. The weight of the negative control mice decrease tremendously where as the weight of crude extracts and fractions treated mice increased gradually. The weight increment in treated mice might be due to the extracts or fractions pharmacological effect that counteract other aspects of malaria illness such as fever, immunosuppression and pain. Mice treated with extracts of *A. integrifolia, C. myricoides, M. azedarach* and *P. vogelii* significantly prevented weight loss in dose dependant fashion. Our finding was consistent with other studies [45–47] where mice treated with extracts of different plants showed weight increment dose dependently.

Malaria-caused anemia occurs due to hemolysis of red blood cells because of destruction of infected and uninfected red blood cells as well as erythropoietic suppression and dyserythropoiesis [48]. The PCVs of malaria-infected mice that were treated with the extracts and fractions were reduced on the 4th day of post infection. The reduction of PCV in the treated mice might be related to the different phytochemical components present in tested plants. Some studies indicated that saponins are known to cause hemolysis by increasing the permeability of plasma membrane of the red blood cells [49]. The methanol extracts and fractions prevented a drastic reduction in PCV in infected mice as compared with that in the negative control. This shows the role of the extracts and fractions in preventing radical anemic conditions that were manifested in mice in negative control due to severe infection. This could be due to the marked decrease in parasite load in the course of infection in mice treated with the extracts and fractions in dose dependant manner. In the untreated mice, the parasite number increased and consequently destroyed more red blood cells and so resulted in marked decrease of hematocrit PCV.

## Conclusions

Acute toxicity test result of methanol crude extracts of the tested plants showed no sign of toxicity in mice treated up to a dose of 2000 mg/kg, supporting their traditional use. In vivo antimalarial test results indicated that crude extracts and fractions of the plant materials showed potent antimalarial activity in dose dependant fashion. Especially, the crude extract and n-butanol and water fractions of *A. integrifolia* showed significant chemosuppression, justifying the local use of the plants to treat malaria. Further in vivo and in vitro investigations are recommended to evaluate, in detail, the antiplasmodial activity and safety of the plants.

### Ethical consideration

The proposal was reviewed and approved by the Institutional Review Board of Aklilu Lemma Institute of Pathobiology, Addis Ababa University. The mice were handled in accordance with national guidelines for handling laboratory animals.

### Availablity of data and materials

Voucher specimens of the tested antimalarial plants were deposited at the National Herbarium of the Addis Ababa University with numbers Bor 9 for *Ajuga integrifolia*, BOR 32 for *Clerodendrum myricoides*, Bor 5 for *Melia azedarach*, BOR 10 for *Peponium vogelii* and BOR 18 for *Premna schimperi*. Antimalarial in vivo tests data were deposited into a computer available at Aklilu Lemma Institute of Pathobiology, Addis Ababa University.
